# Preoperative cerebral oxygenation in high-risk noncardiac surgical patients: an observational study on postoperative mortality and complications

**DOI:** 10.1007/s10877-022-00964-5

**Published:** 2023-01-06

**Authors:** Torsten Baehner, Olaf Perlewitz, Richard K Ellerkmann, Jan Menzenbach, Georg Brand, Marcus Thudium, Markus Velten

**Affiliations:** 1 Department of Anaesthesiology and Intensive Care Medicine, University Hospital Bonn, Bonn, Germany; 2 Department of Anaesthesiology and Intensive Care Medicine, St. Nikolaus Hospital, Andernach, Germany; 3 Department of Anaesthesiology and Intensive Care Medicine Klinikum Dortmund, University Witten/Herdecke, Herdecke, Germany

**Keywords:** Near infrared spectroscopy, Monitoring perioperative, Risk evaluation, Preoperative anesthetic assessment

## Abstract

Near Infrared Spectroscopy (NIRS) has become widely accepted to evaluate regional cerebral oxygen saturation (rScO2), potentially acting as a surrogate parameter of reduced cerebral oxygen delivery or increased consumption. Low preoperative rScO2 is associated with increased postoperative complications after cardiac surgery. However, its universal potential in pre-anesthesia risk assessment remains unclear. Therefore, we investigated whether low preoperative rScO2 is indicative of postoperative complications and associated with poor outcomes in noncardiac surgical patients. We prospectively enrolled 130 patients undergoing high-risk noncardiac surgery. During pre-anesthesia evaluation, baseline rScO2 was recorded with and without oxygen supplementation. The primary endpoint was 30-day mortality, while secondary endpoints were postoperative myocardial injury, respiratory complications, and renal failure. We further evaluated the impact of body position and preoperative hemoglobin (Hb) concentration on rScO2. Of the initially enrolled 130 patients, 126 remained for final analysis. Six (4.76%) patients died within 30 postoperative days. 95 (75.4%) patients were admitted to the ICU. 32 (25.4%) patients suffered from major postoperative complications. There was no significant association between rScO2 and 30-day mortality or secondary endpoints. Oxygen supplementation induced a significant increase of rScO2. Furthermore, Hb concentration correlated with rScO2 values and body position affected rScO2. No significant association between rScO2 values and NYHA, LVEF, or MET classes were observed. Preoperative rScO2 is not associated with postoperative complications in patients undergoing high-risk noncardiac surgery. We speculate that the discriminatory power of NIRS is insufficient due to individual variability of rScO2 values and confounding factors.

## Introduction

A major objective of preoperative anesthetic assessment is the evaluation of cardiac performance and risk. Typically, the medical history is used for this purpose and cardiopulmonary reserve is classified based upon MET stages or NYHA classes. It would, however, be desirable to have an objective parameter which correlates with global cardiopulmonary reserve indicative of perioperative risk. In the case of heart failure, NT-proBNP can be determined to this end, though this necessitates blood sampling and is therefore not immediately available during evaluation [1]. Mixed venous oxygen saturation (SvO2) is useful for determining the ratio of systemic oxygen uptake and oxygen consumption, potentially being used to assess the adequacy of tissue oxygenation [2]. Unfortunately, the determination of mixed venous oxygen saturation requires invasive catheterization of the pulmonary artery, an unreasonably invasive measure for routine preoperative evaluation. It would, therefore, be advantageous to assess a global non-invasive parameter of cardiopulmonary reserve at the point of care. Recent data suggests that regional cerebral oxygen saturation (rScO2), as determined by near-infrared spectroscopy (NIRS), is closely related to SvO2 [3]. The brain has been discussed to act as an index organ for global perfusion [4]. The NIRS signal predominantly represents the venous fraction of the circulation and therefore may indicate oxygen extraction [5, 6]. rScO2 has been shown to depend on cardiac output and global oxygenation and, as it correlates closely with mixed venous oxygen saturation, may be considered as a surrogate parameter of global oxygen delivery [3, 5].

Near Infrared Spectroscopy (NIRS) has become a widely used intraoperative measure to assess rScO2 as a non-invasive marker of cerebral oxygenation, especially during cardiac surgery. rScO2 has also become established in noncardiac surgery involving a risk of cerebral regional hypoperfusion, such as carotid surgery, though its’ value for postoperative outcome is still disputed [7–9]. In addition to an intraoperative use, preoperatively reduced rScO2 was found to be associated with common parameters of cardiac function such as left ventricular function and NYHA Stage in cardiac surgery patients [10]. Moreover, Heringlake and colleagues reported that low preoperative NIRS values were associated with short- and long-term mortality and morbidity after cardiac surgery. The authors concluded that cerebral saturation provides additive information for preoperative risk assessment. It was hypothesized that the brain can be seen as an index organ for perfusion and cerebral oximetry can be used for evaluation thereof [11]. Based upon this assumption, and the observation that patients undergoing cardiac surgery who experience major organ morbidity and mortality present with a decreased baseline rScO2 [10, 12–14], we hypothesized that low preoperative rScO2 is similarly associated with increased postoperative mortality and postoperative complications in noncardiac surgery. We therefore evaluated if NIRS may be used as an additional measure for preoperative risk assessment in this collective.

The aim of the Bonn Cerebral Oxygenation Risk Evaluation (BonnCORE) study was to investigate whether preoperative rScO2 values are associated with increased postoperative morbidity and mortality, with potential use for general preoperative risk assessment in patients undergoing high-risk noncardiac surgery.

## Methods

The BonnCORE trial was performed in accordance with the principles of the Declaration of Helsinki. Approval was granted by the Ethics Committee of University of Bonn, No 162/13. The study was designed as an observational study. Following informed consent, 130 patients undergoing high-risk visceral, vascular, orthopedic, or thoracic surgery were enrolled prospectively. Further inclusion criteria were age between 18 and 89 years and ASA classification I - IV. Only elective patients with high risk surgery according to the modified Johns Hopkins Surgery Risk Classification System were included [15, 16]. Exclusion criteria were cerebral malformations, cerebral malignancies, stroke in medical history, allergies against optodes, cardiac surgery, and pregnancy.

The primary endpoint was defined as 30-day mortality. Secondary endpoints were postoperative myocardial injury (defined as an increase in troponin-T to 150% of normal values), respiratory complications (defined as necessity of non-invasive or invasive ventilation > 48 h), and renal failure (defined by the need for renal replacement therapy or increase of serum creatinine > 2 mg/dl or to more than 200% of preoperative values).

Preoperative evaluation was performed according to institutional standards and ASA guidelines. After screening for inclusion and exclusion criteria, all patients provided informed consent. Upon recruitment, demographic variables were collected and preoperative risk scores were assessed. These include NYHA classification, revised cardiac risk index (rCRI), and metabolic equivalents (MET). All patients were classified into NYHA classes 1 to 4, so the mild NYHA classes may include patients without a history of heart failure.

After preoperative evaluation, NIRS optodes were attached to both sides of the patient’s forehead and rScO2 measurement was performed using the INVOS 5100 Oximeter (Somanetics, Troy, MI, USA). First, the patient was placed in a sitting position breathing ambient air. The arithmetic mean of the left and right forehead measurements was used for further evaluation. Peripheral Pulse oximetry (SpO2) was measured simultaneously. The rScO2 and SpO2 values were documented following a stabilization period of at least 30 s. To exclude impaired pulmonary oxygen uptake as a confounding factor of rScO2, the patient was then asked to inhale oxygen presented at a rate of 2 L per minute. Subsequently, the measurement was repeated and respective values documented. These measurements were repeated in a supine position for the last 52 patients. Outcome data were collected based upon patient medical records or, if the patient was discharged, on the basis of a telephone interview.

### Statistical analysis

Statistical analysis was performed using SPSS version 28 (IBM, Chicago, IL, USA). Continuous variables were expressed as mean ± SD or median (interquartile range). Categorical variables were expressed as the number of participants and percentage. Analysis of variance for categorical variables (NYHA, MET, LVEF) was performed using a one-way ANOVA to test whether rScO2 differed significantly between groups.

We tested rScO2 values with respect to outcome variables (major complications such as myocardial infarction, respiratory failure, acute kidney injury) with Student’s t-test for significance in mean values and the Mann-Whitney-U-Test for significance in median values.

The diagnostic accuracy of rScO2 for predicting the occurrence of endpoint was assessed by receiver operating characteristic (ROC) curve and expressed as area under the curve with 95% CI. Depending on the area under the curve (AUC), prediction performance was classified as poor (0.6–0.7), good (> 0.7), very good (> 0.8), or excellent (> 0.9).

The relationship between rScO2 values and continuous variables (hemoglobin value) was assessed using linear regression, logistic regression was used to examine the relationship between regional cerebral oximetry values and outcome variables. Statistical significance was defined as p < 0.05. All performed tests were two-tailed.

Before we initiated the study, we aimed to perform a sample size calculation. However, the calculation of sample size was associated with considerable uncertainty, as essential parameters were not known with confidence. In contrast to homogeneous populations such as those in cardiac surgery, our collective consisted of a very heterogeneous population. There were not sufficient data available to estimate the distribution of preoperative rScO2 values for this special collective. Furthermore, for this population, there were not enough data available to estimate the effect size. We therefore decided to use the best available and comparable study as a basis for the sample size calculation. This study was the study by Abdelmalak et al. [17]. This approach has natural drawbacks. We therefore performed a post-hoc power analysis to reflect these limitations. The analysis indicates a low effect size, for example for the primary endpoint (ambient air) Cohen’s d -0.17 [95%CI -1, 0.66] and combined endpoint (ambient air) -0.44 [95%CI -0.85, -0.03]. For Cohen’s d 0.2 (low effect size) and primary endpoint (ambient air or oxygen administration), a power of 0.0761561 was calculated. For Cohen’s d 0.5 (medium effect size) and the combined endpoint (ambient air or oxygen administration), a power of 0.6787106 was calculated.

## Results

A total of 130 patients were prospectively enrolled. Two patients were excluded because their rScO2 value could not be measured due to pain preventing them from maintaining a seated position (patient 111 and 113). One patient was excluded because no surgery was performed (patient 18) and another withdrew their consent to the study (patient 56). Finally, 126 patients were available for the final evaluation. For demographic and surgery-related data see Table 1.

**Table 1:** Demographic and surgery related data. Data presented as mean (± SD), or n (%)

Parameter		Value
Patients		126 (100%)
Male sex		80 (63.5%)
Age, years		66.2 (± 10.3)
NYHA	I	34 (27%)
	II	59 (46.8%)
	III	27 (21.4%)
	IV	3 (2.4%)
Coronary heart disease		45 (35.7%)
rCRI	1	16 (12.7%)
	2	41 (32.5%)
	3	30 (23.8%)
	4	39 (31.0%)
Chronic heart failure		36 (28.6%)
COPD		34 (27%)
Chronic kidney disease		37 (29.4%)
*Surgery type *		
Upper GI		27 (21.4%)
Lower GI		20 (15.9%)
Orthopedic		34 (27.0%)
Thoracic		26 (20.6%)
Vascular, n		19 (15.1%)
Disease of tumor as indication for surgery		52 (41.3%)

*COPD* chronic obstructive pulmonary disease, *GI* Gastrointestinal Tract

**Fig. 1:**
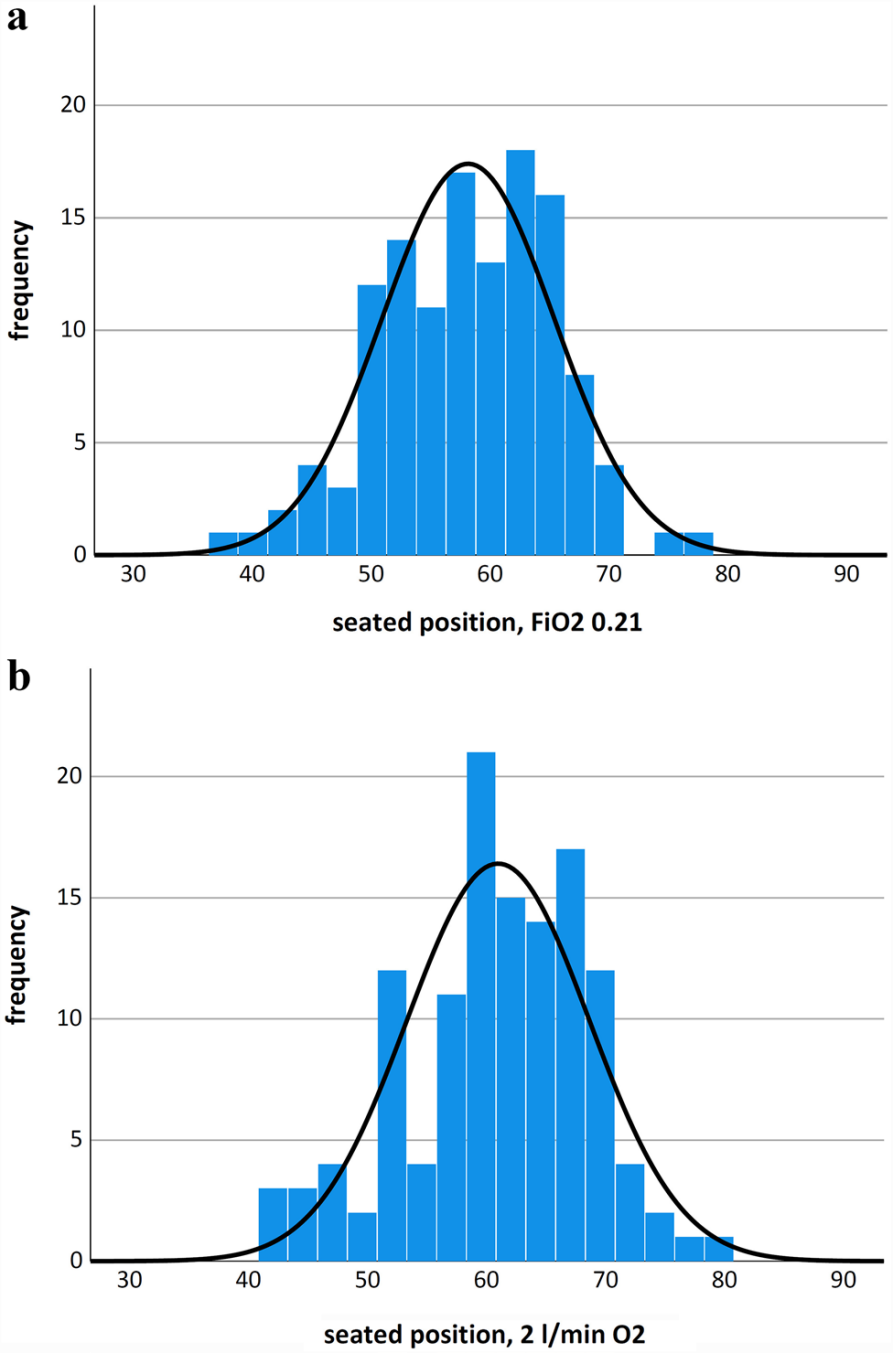
Histograms of rScO2 values in a seated position **a** under ambient air and **b** under administration of 2 L per minute of oxygen. Histogram showing the distribution of rScO2 values and binned frequency counts

The mean rScO2 was 58.1% ± 7.1% in an upright sitting position whilst breathing ambient air. Subsequently, the measurement was repeated with an oxygen administration of 2 L per minute. We found a significant rScO2 increase of 2.82% ± 2.18% (p < 0.001) under oxygen administration, with the mean rScO2 raised to 60.9% ± 7.6%. In both measurements, a normal distribution could be observed (see Fig. 1).

Of the 126 patients in the study group, 6 (4.76%) died within the first 30 postoperative days. 95 (75.4%) patients were admitted to an intensive care unit because of their preoperative status or due to the intraoperative course of surgery.

Some patients suffered from serious postoperative complications, we therefore compared the preoperative rScO2 values of the patients with and without such complications. There was no significant difference in preoperative rScO2 values with respect to primary or secondary endpoints. Details of these postoperative complications and associated rScO2 values are presented in Table 2. Statistical analysis did not indicate a significant correlation between rScO2 and postoperative 30-day-mortality (p = 0.682) or other singular secondary endpoints, such as myocardial infarction (n = 8, p = 0.621), respiratory failure (n = 19, p = 0.061) or acute kidney injury (n = 15, p = 0.819).

**Table 2:** Primary and secondary endpoints and rScO2 with **a** ambient air breathing or **b** breathing 2 L per minute oxygen. Data presented as mean (± standard deviation). 30-day mortality and major postoperative complications myocardial infarction, respiratory failure, and acute kidney failure

(a)	n (%)	rScO2(FiO2 0.21)	Reference	p
All	126 (100)	58.1 (±7.2)	–	–
Primary endpoint 30-day Mortality	6 (4.8)	56.9 (±8.2)	58.2 (±7.2)	0.682
*Secondary endpoints *				
Myocardial infarction	8 (6.3)	56.9 (±6.2)	58.2 (±7.3)	0.621
Respiratory failure	30 (23.8)	55.9 (±6.6)	58.8 (±7.3)	0.061
Acute kidney failure	15 (11.9)	57.7 (±6.0)	58.2 (±7.4)	0.819

(b)	n (%)	rScO2(2 L/minO2)	Reference	p
All	126 (100)	60.9 (±7.7)	–	–
Primary endpoint 30-day Mortality	6 (4.8)	59.2 (±9.3)	61.0 (±7.6)	0.567
*Secondary endpoints*				
Myocardial infarction	8 (6.3)	60.0 (±7.4)	60.9 (±7.7)	0.745
Respiratory failure	30 (23.8)	58.8 (±6.9)	61.6 (±7.8)	0.085
Acute kidney failure	15 (11.9)	60.8 (±6.4)	60.9 (±7.8)	0.963

**Fig. 2:**
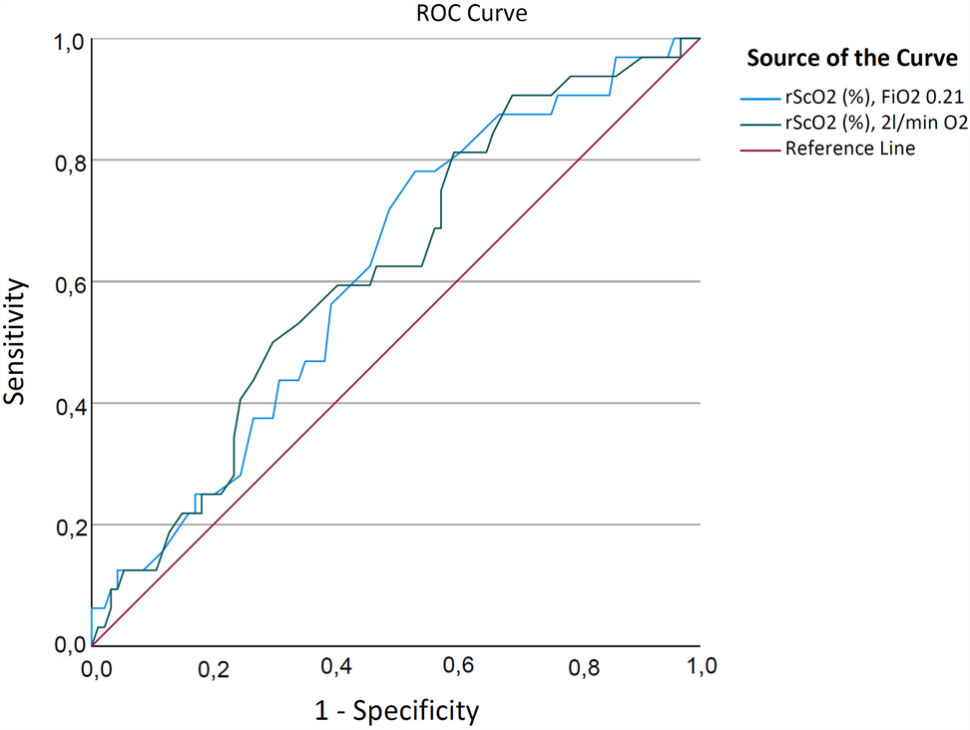
Receiver operating curve analysis of preoperative cerebral oxygen saturation rScO2 and the combined endpoint of all evaluated complications. Blue ROC curve breathing ambient air (p = 0.05) and red ROC curve breathing 2 L per minute oxygen (p = 0.047)

A subsequent analysis combining all primary and secondary endpoints revealed a statistically significant lower rScO2 baseline in patients with this combined endpoint. The 32 (25%) patients with major complications revealed a statistically significant lower preoperative rScO2 value of 55.8% ± 7.2% vs. 58.9% ±7.1% whilst breathing ambient air (p = 0.035). This statistical significance difference was not sustained under supplementation with 2 L/min oxygen (58.8%, ± 7.5% vs. 61.7%, ± 7.6%, p = 0.063). Receiver operating characteristic (ROC) of rScO2 under ambient air and 30-day mortality revealed an area under the curve (AUC) of 0.616 (95% CI, 0.508–0.723; p = 0.051). ROC curve of rScO2 under 2 L/min oxygen supplementation and 30-day mortality revealed an area under the curve (AUC) of 0.618 (95% CI, 0.509–0.726; p = 0.047) (see Fig. 2).

**Fig. 3:**
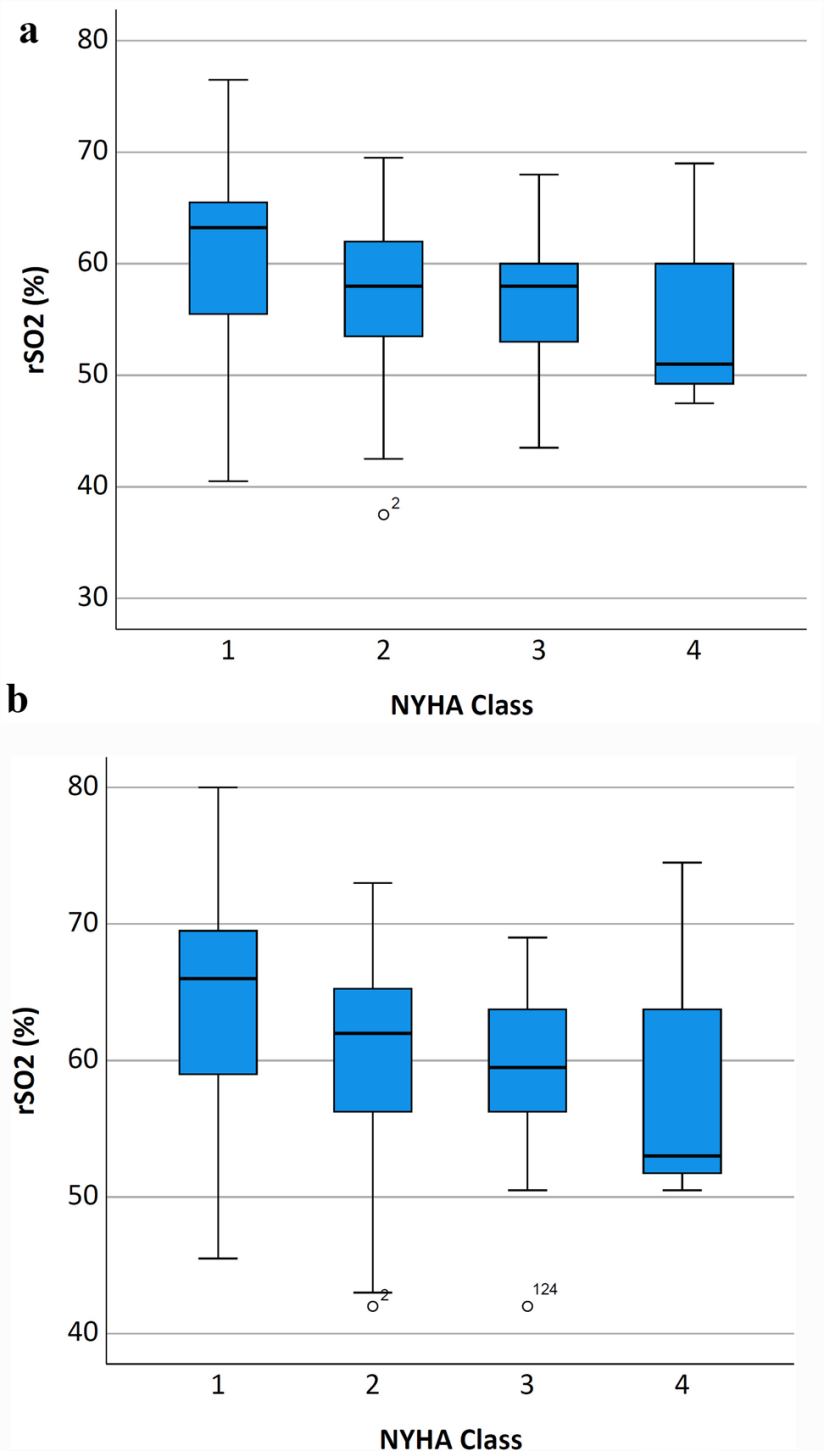
Boxplots of rScO2 distribution comparing **a** breathing ambient air or **b** breathing 2 L per minute oxygen regarding increasing NYHA Class. The box represents 25th and 75th percentiles; the range between them is the interquartile range. Within the box, the bold line represents the median. The whiskers (extensions from the box) indicate the lowest and highest value no further than 1.5 times the interquartile range. Outliers (values beyond whiskers) are shown as dots

**Fig. 4:**
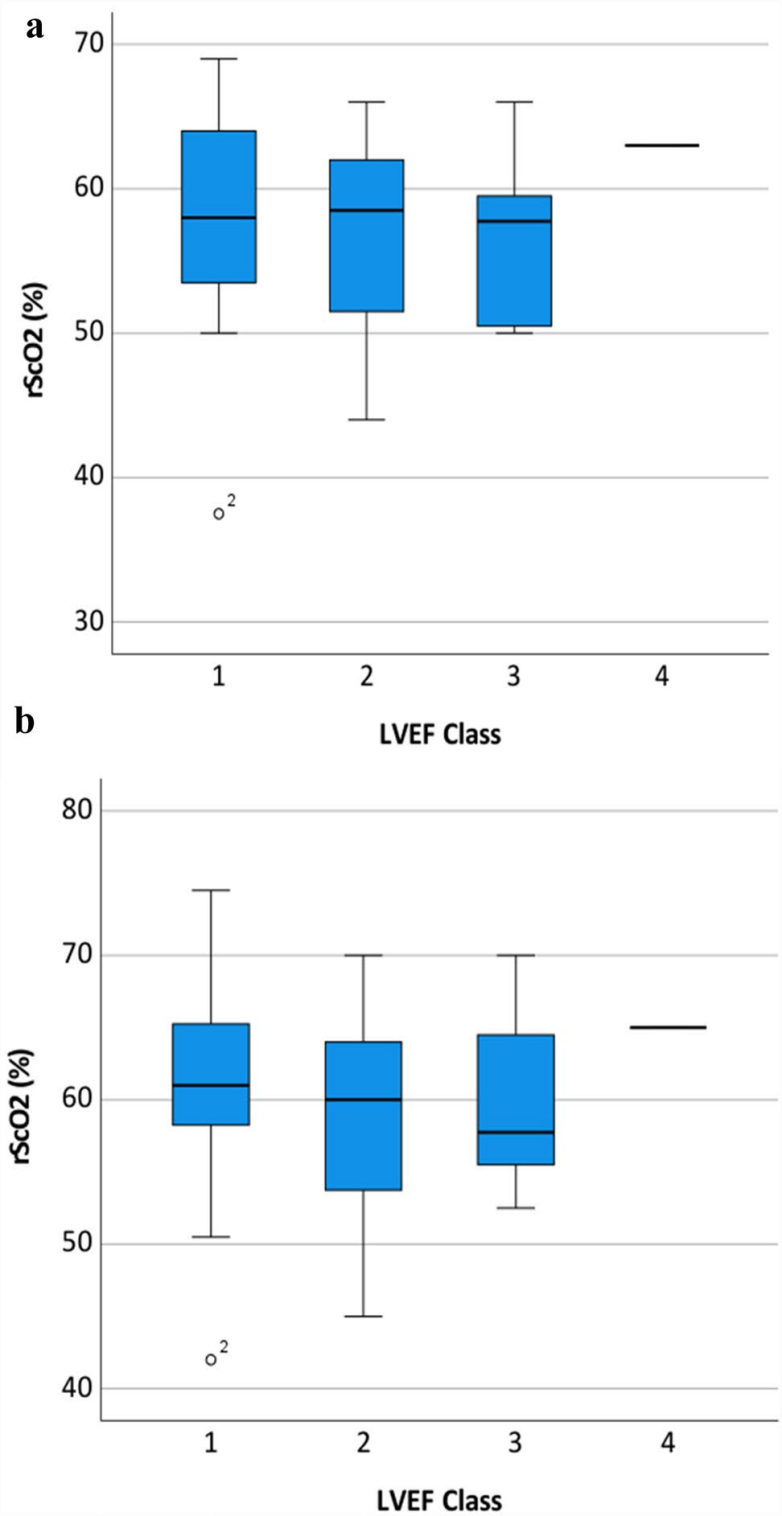
Boxplots of rScO2 distribution comparing **a** breathing ambient air or **b** breathing 2 L per minute oxygen regarding LVEF Class [31]. Left ventricular function Class (1) Normal = LVEF 50–70% (midpoint 60%), (2) Mild dysfunction = LVEF 40–49% (midpoint 45%), (3) Moderate dysfunction = LVEF 30–39% (midpoint 35%), (4) Severe dysfunction = LVEF less than 30%. The box represents 25th and 75th percentiles; the range between them is the interquartile range. Within the box, the bold line represents the median. The whiskers (extensions from the box) indicate the lowest and highest value no further than 1.5 times the interquartile range. Outliers (values beyond whiskers) are shown as dots

Additionally, rScO2 values were related to established parameters of cardiopulmonary function. We were able to show a trend towards lower rScO2 values with increasing NYHA class under ambient air as well as after oxygen supplementation respectively, though this effect was not significant (p = 0.074 and 0.107, see Fig. 3). Regarding general functional performance represented by the metabolic equivalent (MET), we found no difference in rScO2 values in different MET groups (ambient air p = 0.207 and oxygen supplementation p = 0.263). With respect to cardiac function represented by left ventricular ejection fraction, there was also no significant difference of rScO2 in different LVEF groups (under ambient air p = 0.549 and oxygen supplementation 0.578, see Fig. 4).

**Fig. 5:**
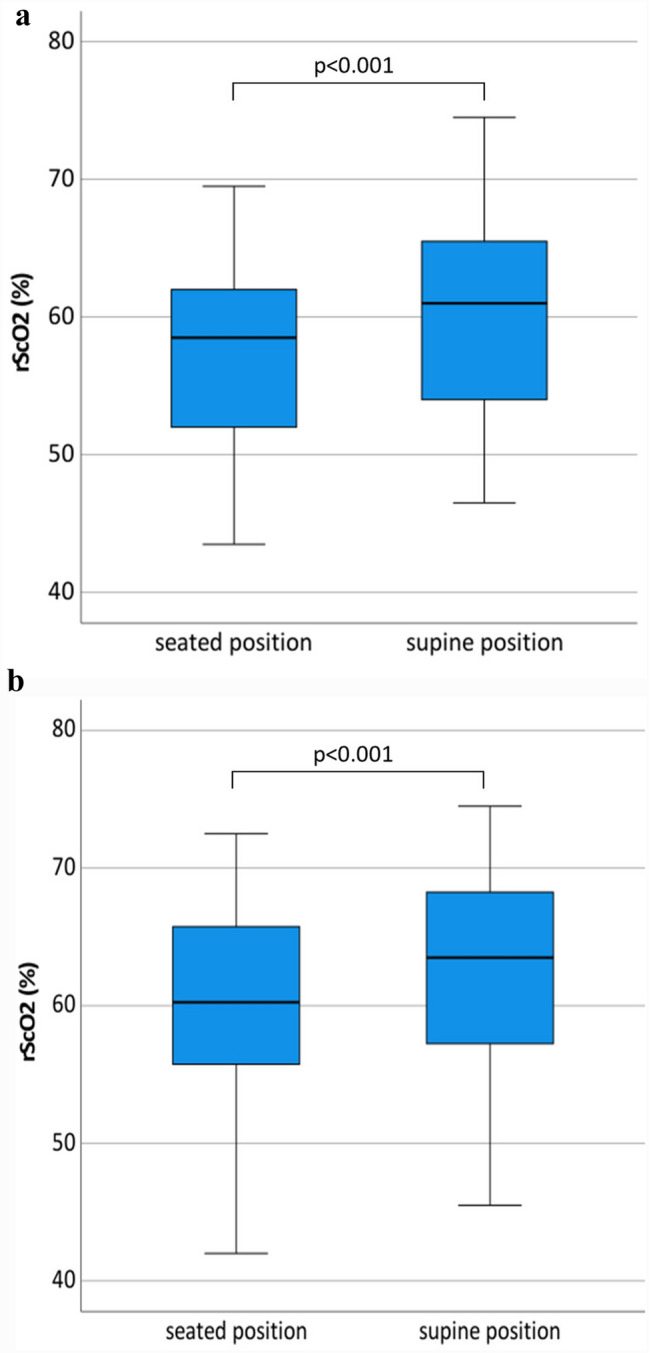
Boxplots of rScO2 distribution comparing **a** breathing ambient air or **b** breathing 2 L per minute oxygen regarding body position. On the left side of the diagram the boxplot represents the rScO2 distribution of the patient in the seated position, subsequently the measurement was repeated in the supine position, shown on the right side of the diagram. The box represents 25th and 75th percentiles; the range between them is the interquartile range. Within the box, the bold line represents the median. The whiskers (extensions from the box) indicate the lowest and highest value no further than 1.5 times the interquartile range

In a subgroup of 52 individuals (41.3%), we repeated rScO2 measurements in a supine position under ambient air and oxygen supplementation. This revealed a significant increase of mean rScO2 values caused by the transition of body position from seated to supine under ambient air (57.1 ± 6.8 vs. 59.9 ± 7.3) and under oxygen supplementation (59.7 ± 7.7 vs. 62.1 ± 7.5, p < 0.001), as shown by Fig. 5.

**Fig. 6:**
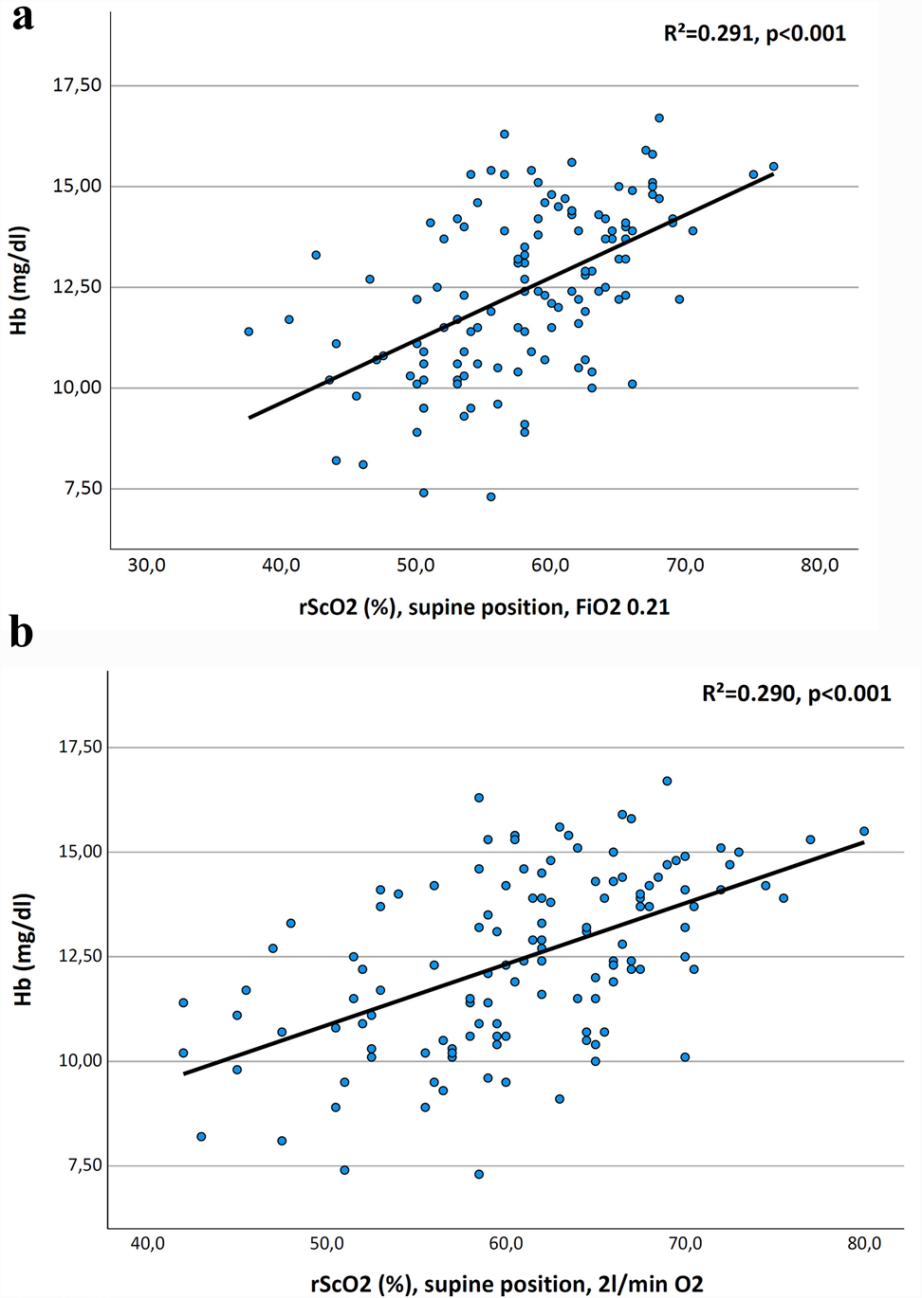
Correlation of rScO2 to preoperative hemoglobin value in **a** seated position and room air **b** in seated position under oxygen supplementation. R² Pearson correlations coefficient, p level of significance

Hemoglobin is known as a confounding factor of rScO2. The mean preoperative hemoglobin value was 12.4 ± 2.1 mg/dl. Statistical analyses revealed a correlation between preoperative hemoglobin concentration and preoperative rScO2 (Pearson correlations coefficient, rSO2 FiO2 0.21 0.540, p < 0.001, Pearson correlations coefficient rSO2 2 L/min O2 0.538, p < 0.001) (see Fig. 6).

## Discussion

Alongside the demographic ageing of our population, we are seeing an increase in patients at risk for perioperative complications requiring detailed preoperative risk assessment. Risk evaluation is time consuming and established scores predicting postoperative mortality are complex and to some degree subjective, resulting in a certain degree of variability [18, 19].

Therefore, the introduction of a simple, objective, and non-invasive method providing a reliable value for risk stratification, would be of great advantage to preoperative assessments, even if the provided value would only be indicative or additive. We hypothesized that the brain can be considered an index organ of global perfusion and assessment of cerebral oxygenation using NIRS can be used for risk stratification [11].

Multiple studies have proven low baseline rScO2 to be predictive of postoperative complications in cardiac surgery patients, resulting in a recent recommendation of the American Society for Enhanced Recovery and Perioperative Quality Initiative. It now includes the interpretation of perioperative cerebral oximetry measurements in the context of a preinduction baseline value in cardiac surgery patients [10, 12–14, 20].

Therefore, the question arises whether this aspect could also be transferred to a noncardiac surgical collective. To the best of our knowledge, this is the first study investigating whether preoperative baseline of rScO2 using near-infrared spectroscopy can be used to predict postoperative complications and mortality in noncardiac surgical patients.

In contrast to the cardiac surgical population our study did not reveal an association between low preoperative rScO2 values measured by NIRS and postoperative mortality and morbidity [10]. In the cardiac surgical population, Heringlake et al. reported significantly lower preoperative rScO2 with increasing NYHA stage. They concluded that rScO2 baseline values reflect the severity of cardiopulmonary dysfunction and recommend the use of preoperative rScO2 values for risk stratification. In the general surgical population of this study, we found a similar trend of decreasing rScO2 values with increasing NYHA stage, though this did not prove statistically significant. However, it is important to note the difference between the two study populations. Heringlake’s patients may be more homogenous, consisting exclusively of cardiac surgery patients, had a higher mean rScO2 of 62% compared to our patients with a mean of 58.1%. Additionally, rScO2 range was considerably broader in our population (38–77% under ambient air and 42–80% under 2 L/min O2) than those of Heringlake et al., which ranged from 56 to 67% under ambient air and 61–71% when breathing oxygen-enriched air and revealed significantly lower hemoglobin values (12.4 vs. 13.5 mg/dl) [10]. Hemoglobin concentration has a significant impact on rScO2 according to the literature and our findings (see below). This may reflect the diversity of pathologies in our heterogeneous collective, potentially caused by patients with severe pulmonary impairment or anemia that may not be present in a cardiac surgery setting.

Ghosal et al. reported that a higher rScO2 at baseline is associated with lower 30-day mortality (OR 0.94, CI 0.888–0.995) for every 1% increase of rScO2 in a retrospective review of 210 continuous flow LVAD patients [12]. However, this study did not report an association between baseline rScO2 and secondary outcomes (e.g. cardiocerebral events, length of hospital stay, and intensive care unit stay). Sun et al. analyzed rScO2 levels prior to induction of anesthesia in 2097 patients undergoing cardiovascular surgery [14]. Using an rScO2 cut off value of 60%, the patients below the cut off level exhibited significantly higher mortality. The rScO2 cut off value below 60% additionally correlated with accepted cardiac surgery mortality scores. While this supports the theory that rScO2 may have a role in preoperative risk evaluation in cardiac surgery, we found no association with single variables of morbidity in our noncardiac surgery collective. However, the subsequent analysis of a combined outcome including all adverse events revealed significantly lower rScO2 values in patients with adverse events. Despite this significant difference between the groups with respect to the baseline rScO2 and the combined endpoint, ROC analysis indicates a very poor sensitivity and specificity. The AUC of 0.62 barely exceeds the AUC limit of 0.6, under which the test has to be completely rejected.

rScO2 is influenced by several confounders that affect baseline variability. While cerebral NIRS theoretically measures regional oxygenation of the anterior cortex, the influence of extracranial tissues has also been described [21]. Furthermore, we identified additional confounding variables that affect baseline variability. By changing the patient’s body position from seated to supine, the cerebral blood volume and the ratio of arterial to venous intracranial blood can be changed. We were able to demonstrate the effect of body position on rScO2 in a subgroup analysis and found a highly significant raise of rScO2 following the change from a seated to a supine position and consistent with previous studies a close correlation between the baseline rScO2 values and preoperative hemoglobin concentration indicating that NIRS is sensitive to hemoglobin values [22].

Recently, it was discussed that age-related microvascular dysfunction could be assessed by rScO2. Rosenberry et al. investigated postocclusion tissue oxygenation and recovery kinetics using rScO2 [23]. The group found an age correlation of rScO2 reperfusion kinetics. Holmes et al. investigated the balance of oxygen supply and consumption during physical activity using rScO2 [24]. For this purpose, rScO2 was performed at the medial gastrocnemius during a 6 min walk test in patients in different age groups. This group also found a strong correlation of rScO2 with increasing age. In addition to age-dependence, other factors influencing rScO2 have been described. Valencia et al. [25], for example, found a correlation between ASA status and baseline rScO2 in a small observational post-hoc analysis. Moreover, other correlations such as a dependence on body weight, height and renal function have also been described [25–27]. Therefore, it can be concluded that there are several factors that influence the rScO2 value, but a direct linear correlation has only been demonstrated for a limited number of factors. It is conceivable that these confounding factors could be considered in a scientific approach, e.g., by propensity score matching. The present study, however, aimed for a simple apparative parameter to be used bedside, e.g. during preanesthetic evaluation. The need to correct for many confounding factors makes this parameter unsuitable for this purpose.

In addition to the method-specific confounders, there are other confounders of the operative and postoperative period which have an impact on the postoperative outcome. Thus, the difficult-to-stratify surgical impact factor as a relevant determinant of outcome [28] as well as the difficult-to-measure variable of fluctuating quality of postoperative care [29, 30]. Another issue is the heterogeneity of our study population, in contrast to the more homogenous collective in cardiac surgery. Differing surgical specialties may also limit comparability as, for example, orthopedic procedures can result in other morbidities than thoracic or upper abdominal procedures.

Although rScO2 as measured by NIRS has become a safe and established non-invasive method, precisely detecting intraindividual trend changes of cerebral oxygenation and perfusion, there is a marked variability of baseline values between individuals. From our point of view, this is the main drawback to this method and its’ widespread use for risk stratification. Unfortunately, this variability cannot be attributed to cardiopulmonary function or the ratio of oxygen supply to oxygen consumption alone. Thus, this method has its limitations in the assessment of cardiopulmonary function and the ratio of oxygen supply to oxygen consumption and limitations of our study need to be addressed. The range of rScO2 values in our patients was very broad and the difference of rScO2 values between patients with postoperative major complications to those without was relatively small. Furthermore, the ROC curve exhibited poor sensitivity and specificity for the combined endpoint.

One limitation that need to be addressed is the sample size calculation. Since the present investigation is the first of its kind in non-cardiac surgery patients, key parameters for a priori calculation were not available. Therefore, we performed a post-hoc power analysis for both the primary endpoint (30-day mortality) and the composite endpoint. To this end, we determined the observed effect size with a 95% confidence interval and calculated post-hoc power for the observed sample sizes in the study groups. In addition, we calculated the test power for fixed (not study-specific) Cohen’s d values (effect sizes). Due to the observed small effect size (Cohen’s d), the power of the method regarding the primary endpoint was expected to be limited. It should be noted, that the post-hoc analysis revealed an acceptable power of 0.678 for the combined endpoint. However, the results of the post-hoc power analysis confirm our key message. The large variability of the rScO2 and the small difference of the mean substantially limited power of the method for the aimed application.

In conclusion, our data revealed that preoperative rScO2 was not useful for the prediction of 30-day mortality or any single postoperative complication in patients undergoing noncardiac high-risk surgery. However, there was a statistical significant difference in rScO2 for the combined endpoint, occurrence of any complication, without a “clear-cut” association between preoperative rScO2 and postoperative complications, indicating that baseline rScO2 evaluation has a potential for preoperative risk stratification in non-cardiac patients and further investigations are required.
